# Diet-derived circulating antioxidants and risk of epilepsy: a Mendelian randomization study

**DOI:** 10.3389/fneur.2024.1422409

**Published:** 2024-07-05

**Authors:** Shicun Huang, Yingqi Chen, Yiqing Wang, Shengjie Pan, Yeting Lu, Wei Gao, Xiaowei Hu, Qi Fang

**Affiliations:** ^1^Department of Neurology, First Affiliated Hospital of Soochow University, Suzhou, China; ^2^Department of Neurology, Suzhou Hospital of Traditional Chinese Medicine, Suzhou, China

**Keywords:** diet-derived circulating antioxidants, zinc, epilepsy, Mendelian randomization, oxidative stress

## Abstract

**Background:**

Previous studies suggest a link between diet-derived circulating antioxidants and epilepsy, but the causal relationship is unclear. This study aims to investigate the causal effect of these antioxidants on epilepsy.

**Methods:**

To assess the causal link between dietary antioxidants and epilepsy risk, we conducted a two-sample Mendelian randomization (MR) analysis. This involved examining antioxidants such as zinc, selenium, α- and γ-tocopherol, vitamin A (retinol), vitamin C (ascorbate), and vitamin E (α-tocopherol). We utilized instrumental variables (IVs) which were genetic variations highly associated with these commonly used antioxidants. Exposure data were sourced from a comprehensive genome-wide association study (GWAS). We aggregated data from the International League Against Epilepsy (ILAE) Consortium sample, which included various types of epilepsy, as an outcome variable. Finally, we applied the inverse variance weighting method and conducted sensitivity analyses for further validation.

**Results:**

Based on the primary MR estimates and subsequent sensitivity analyses, the inverse variance weighting (IVW) method revealed that a genetically predicted increase in zinc per standard deviation was positively associated with three types of epilepsy. This includes all types of epilepsy (OR = 1.06, 95% CI: 1.02–1.11, *p* = 0.008), generalized epilepsy (OR = 1.13, 95% CI: 1.01–1.25, *p* = 0.030), and focal epilepsy (documented hippocampal sclerosis) (OR = 1.01, 95% CI: 1.00–1.02, *p* = 0.025). However, there is no evidence indicating that other antioxidants obtained from the diet affect the increase of epilepsy either positively or negatively.

**Conclusion:**

Our research indicates that the risk of developing epilepsy may be directly linked to the genetic prediction of zinc, whereas no such association was found for other antioxidants.

## 1 Introduction

Epilepsy is a brain disorder defined by at least two unprovoked seizures over 24 h apart, one unprovoked seizure with a high recurrence risk, or an epilepsy syndrome diagnosis; it is considered resolved in individuals who have been seizure-free for 10 years and off medication for the last 5 years (1). As the most widespread severe chronic neurological condition, epilepsy affects 70 million people worldwide and has significant cognitive, social, psychological, and economic impacts (2, 3). Despite the availability of many new ASDs, the outcomes for newly treated epilepsy and the likelihood of drug resistance remain largely unchanged from earlier studies (4). Therefore, further investigation into the underlying mechanisms is urgently needed to develop new therapeutic approaches. Clinical and experimental studies indicate that oxidative stress is both a cause and a consequence of epilepsy progression (5, 6). The onset of epilepsy is linked to oxidative stress and the overproduction of reactive oxygen species (ROS) (7–9). Epileptic seizures induce oxidative stress, leading to further neuronal damage and triggering a chain reaction of subsequent seizures (10), with both factors interacting and influencing each other. Unfortunately, there is currently no effective medication available to reduce neuronal death by modulating oxidative stress and thereby improve epilepsy.

Many compounds with antioxidant properties have been extensively researched for their antiepileptogenic therapeutic potential, given the role of antioxidant defense systems in neutralizing the increased generation of reactive oxygen species (ROS) during seizures (11–13). Antioxidants found in dietary supplements (14, 15), such as vitamins E and C, vitamin A, carotenoids, zinc and selenium, attract special attention due to their accessibility and ease of intake modification. However, the impact of these diet-derived circulating antioxidants on epilepsy remains controversial. While some reports suggest these antioxidants are safe and without adverse effects, others indicate potential impacts on the central nervous system and an increased risk of seizures associated with their use (14, 16–18). Despite the global popularity of dietary supplements, including among patients with epilepsy (ranging from 10 to 56%) (14, 15, 19–21), evidence supporting their beneficial effects in epilepsy remains lacking.

The existing results are generally inconsistent, leading to uncertainty about the association between diet-derived circulating antioxidants and the risk of epilepsy. Current studies are primarily in early research stages, such as animal experiments, and there is limited clinical evidence. To address these limitations and improve the study design, Mendelian randomization (MR) analysis was incorporated (22). Genetic variation was used as an instrumental variable (IV) to establish robust causal inferences regarding the relationship between levels of common diet-derived antioxidants and the occurrence of epilepsy. This methodology helps minimize biases from confounding factors and reverse causality. By using genetic variations as proxies for antioxidant levels, this approach may facilitate a deeper understanding of preventive strategies for epilepsy and related conditions.

## 2 Methods

### 2.1 Study design

**Figure 1:**
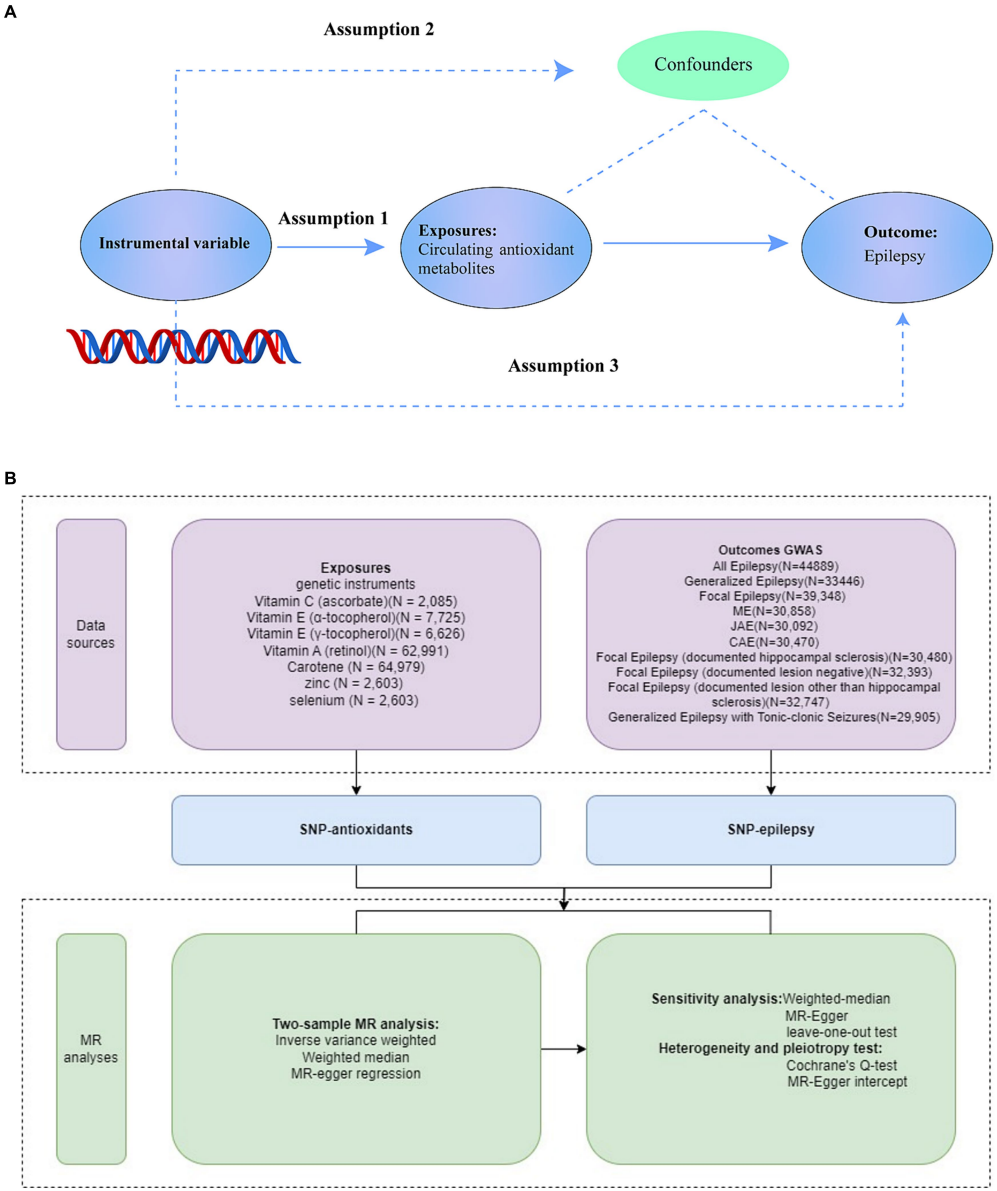
Overview of the study design **(A)**, Study design. **(B)**, Data Sources of Exposures, Outcomes, and MR analyses. SNPs for dietary antioxidants (vitamin A, vitamin C, vitamin E, carotene, zinc, and selenium) were identified as genetic instrumental variables. Summary statistics for epilepsy associations were obtained from GWAS Catalog. For each exposure, MR analyses (primary analysis using inverse-variance weighted (IVW), weighted median, MR-Egger regression, and sensitivity analyses using Cochrane’s test, Egger intercept, and leave-one-out test) were performed. GWAS, genome-wide association study; SNP, single-nucleotide polymorphism; MR, Mendelian randomization.

Figure 1 provides a schematic overview of the current investigation. We first obtained relevant genetic variations from extensive genome-wide association studies (GWASs) for carotene, zinc, selenium, vitamin A (retinol), vitamin C (ascorbate), and vitamin E (α-tocopherol). Summary data related to epilepsy were taken from the GWAS Catalog. Using several sensitivity analyses and a two-sample MR study, we assessed the causal links between diet-derived antioxidants and epilepsy (23). The MR analysis relies on three key assumptions: (1) the genetic variants must be associated with the exposure, (2) the genetic variants must influence the outcome through the exposure, and (3) the genetic variants must be independent of all confounding factors. Each original study received informed consent and ethical approval, and all data used in this investigation are publicly available.

### 2.2 Selection of genetic instrumental variables

The present study considered seven primary dietary-derived antioxidants: vitamin A (retinol), vitamin C (ascorbate) (24), vitamin E (α-tocopherol) (24), vitamin E (γ-tocopherol) (24), carotene, zinc (25), and selenium (25). Based on extensive GWASs, single-nucleotide polymorphisms (SNPs) associated with these diet-derived antioxidants were identified as instrumental variables (IVs), using the following criteria: *p* < 5 × 10^–6^, linkage disequilibrium (LD) with *r*^2^ < 0.001, and LD distance >10,000 kb. An *F*-statistic exceeding 10 signified a robust association between the IVs and antioxidants, with the strength of this correlation evaluated between SNPs and diet-derived antioxidants. The specific SNPs linked to antioxidants are detailed in Supplementary Table S1.

### 2.3 Outcome data sets

Through a meta-analysis (*N* case = 15,212, *N* control = 29,677) conducted by the International League Against Epilepsy Consortium on Complex Epilepsies(ILAE) in 2018 (26), we obtained GWAS summary statistics for epilepsy. Seizures and epileptic disorders were diagnosed following the International League Against Epilepsy (ILAE)’s classification and nomenclature rules. Harmonization techniques were used to align SNP effect sizes and remove strand mismatches. Summary statistics are available in Table 1. To our knowledge, there was no sample overlap between the outcome and exposure GWASs.

**Table 1:** Characteristics of diet-derived antioxidants and epilepsy datasets.

Trait	Year	Author	Population	SNP	Unit	Sample Size	PMID	Dataset
**Exposure (circulating antioxidants metabolites)**								
Vitamin A (retinol)	2018	Ben Elsworth	European	9	SD	62,991	NA	ukb-b-17406
Vitamin C (ascorbate)	2014	Shin	European	10	log10-transformed metabolites concentration	2,085	24816252	met-a-348
Vitamin E (α-tocopherol)	2014	Shin	European	5	log10-transformed metabolites concentration	7,725	24816252	met-a-340
Vitamin E (γ-tocopherol)	2014	Shin	European	7	log10-transformed metabolites concentration	6,226	24816252	met-a-571
Carotene	2018	Ben Elsworth	European	16	SD	64,979	NA	ukb-b-16202
Zinc	2013	Evans	European	8	SD	2,603	23720494	ieu-a-1079
Selenium	2013	Evans	European	6	SD	2,603	23720494	ieu-a-1077

Outcomes (epilepsy)	Year	Author	Population	Cases	controls	Sample Size	PMID	Dataset
All epilepsy	2018	Abou-Khalil B	Mixed	15,212	29,677	44,889	30531953	ieu-b-8
Generalized epilepsy	2018	Abou-Khalil B	Mixed	3,769	29,677	33,446	30531953	ieu-b-9
Focal epilepsy	2018	Abou-Khalil B	Mixed	9,671	29,677	39,348	30531953	ieu-b-10
JME	2018	Abou-Khalil B	Mixed	1,181	29,677	30,858	30531953	ieu-b-17
JAE	2018	Abou-Khalil B	Mixed	415	29,677	30,092	30531953	ieu-b-12
CAE	2018	Abou-Khalil B	Mixed	793	29,677	30,470	30531953	ieu-b-13
Focal epilepsy (documented hippocampal sclerosis)	2018	Abou-Khalil B	Mixed	803	29,677	30,480	30531953	ieu-b-14
Focal epilepsy (documented lesion negative)	2018	Abou-Khalil B	Mixed	2,716	29,677	32,393	30531953	ieu-b-11
Focal epilepsy (documented lesion other than hippocampal sclerosis)	2018	Abou-Khalil B	Mixed	3,070	29,677	32,747	30531953	ieu-b-15
Generalized epilepsy with tonic–clonic seizures	2018	Abou-Khalil B	Mixed	228	29,677	29,905	30531953	ieu-b-16

SNP, single-nucleotide polymorphism.

### 2.4 Statistical analysis

#### 2.4.1 Two-sample Mendelian randomization analysis

In the primary MR analysis, inverse-variance weighted (IVW) regression was utilized, assuming no flawed genetic instruments such as directional pleiotropy (27). A fixed-effect IVW meta-analysis of the Wald ratios was performed, calculating the gene-outcome (log odds ratio) divided by the gene-exposure correlations for each instrumental variable, to derive the mean impact estimate from each outcome database individually (28). The results are presented as odds ratios (ORs), indicating the impact of antioxidants on epilepsy risk, either based on standard deviation (for retinol, carotene, zinc, and selenium) or natural log-transformed levels (for ascorbate, α-tocopherol, and γ-tocopherol). These ORs evaluate the causal relationship between the exposure and the outcome, assuming the MR assumptions are met.

#### 2.4.2 Sensitivity analysis

To assess heterogeneity and horizontal pleiotropy, this study used Egger regression intercepts (29) and Cochrane’s *Q* test (30) for sensitivity analysis. Cochrane’s *Q* test evaluated heterogeneity among instrumental factors, while potential horizontal pleiotropy was examined using MR-Egger intercept tests. Additionally, a leave-one-out test was conducted by iteratively removing each SNP and re-estimating the MR data. Considering consistency across all MR approaches, the IVW method was chosen as the primary estimate for causal effects based on these analyses.

The study and analysis were performed using R-version 4.3.2, utilizing the MR and “TwoSampleMR packages” (31, 32).

## 3 Results

### 3.1 Exposure and outcome

Table 1 displays the characteristics of participants in the epilepsy and antioxidants datasets. Specifics on SNPs linked to vitamin A (retinol), vitamin C (ascorbate), vitamin E (α-tocopherol), vitamin E (γ-tocopherol), carotene, zinc, and selenium are provided in Supplementary Table S1. A total of 59 SNPs were included as IVs for each of the seven antioxidants. The *F*-statistic for each genetic tool used in this investigation exceeded 10.

### 3.2 Main findings

**Figure 2:**
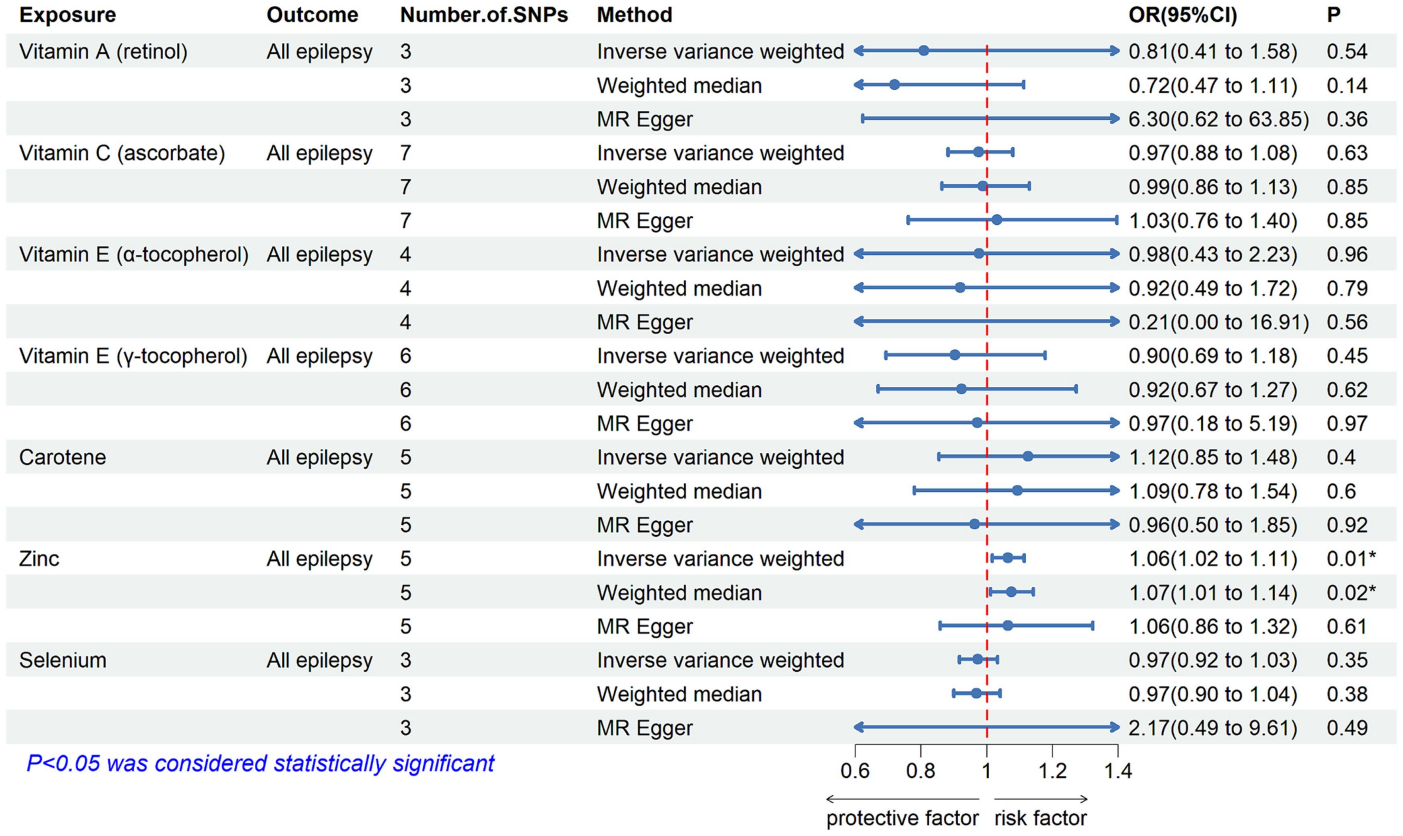
The association between genetically determined diet-derived antioxidants and the risk of all types of epilepsy. Estimated ORs (odds ratio) for the effect of per unit increase in vitamin A (retinol), vitamin C (ascorbate), vitamin E (α-tocopherol), vitamin E (γ-tocopherol), carotene, zinc, and selenium on all types of epilepsy from an inverse-variance weighted (IVW) analysis. *p-value <0.05.

**Figure 3:**
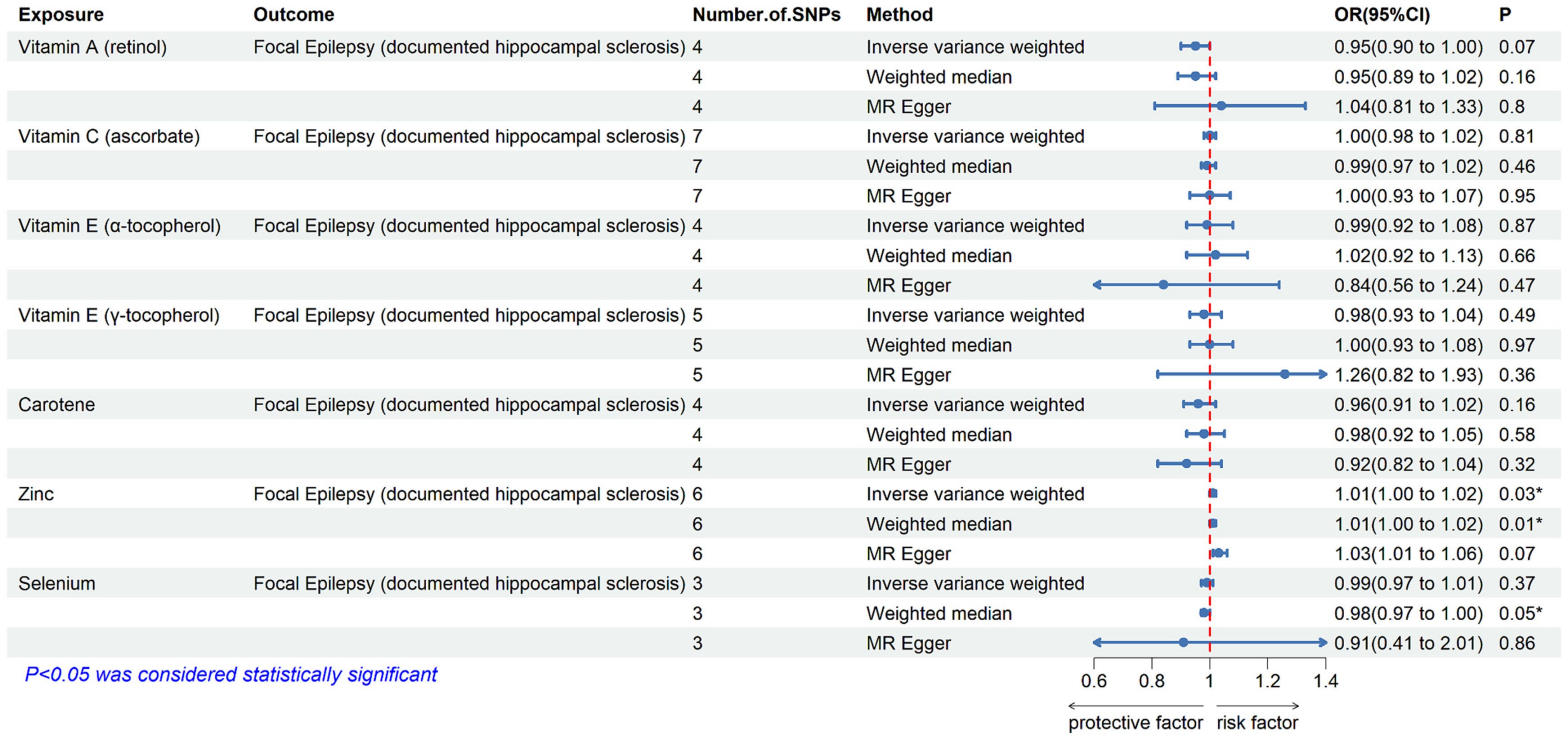
The association between genetically determined diet-derived antioxidants and the risk of generalized epilepsy. Estimated ORs (odds ratio) for the effect of per unit increase in vitamin A (retinol), vitamin C (ascorbate), vitamin E (α-tocopherol), vitamin E (γ-tocopherol), carotene, zinc, and selenium on generalized epilepsy from an inverse-variance weighted (IVW) analysis. *p-value <0.05.

**Figure 4:**
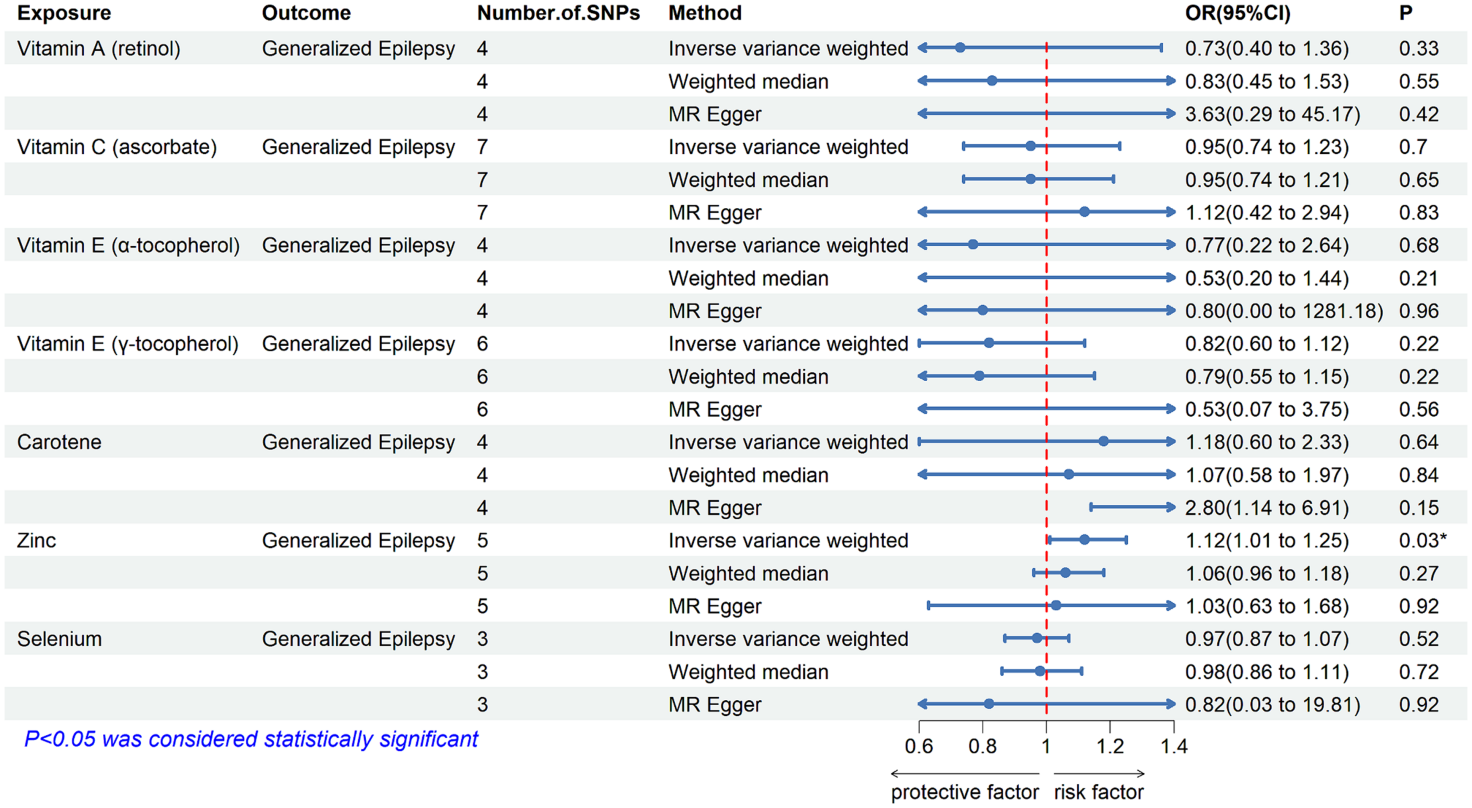
The association between genetically determined diet-derived antioxidants and the risk of focal epilepsy (documented hippocampal sclerosis). Estimated ORs (odds ratio) for the effect of per unit increase in vitamin A (retinol), vitamin C (ascorbate), vitamin E (α-tocopherol), vitamin E (γ-tocopherol), carotene, zinc, and selenium on focal epilepsy (documented hippocampal sclerosis) from an inverse-variance weighted (IVW) analysis. *p-value <0.05.

Table 2 displays the results of the MR analysis investigating the impact of zinc on epilepsy. The findings suggest a causal relationship between genetically determined blood zinc levels and three distinct types of epilepsy. This association is observed across all epilepsy types (OR = 1.06, 95% CI: 1.02–1.11, *p* = 0.008), generalized epilepsy (OR = 1.13, 95% CI: 1.01–1.25, *p* = 0.030), and focal epilepsy (documented hippocampal sclerosis) (OR = 1.01, 95% CI: 1.00–1.02, *p* = 0.025). Elevated blood zinc levels are linked to an increased risk of these three seizure types. Subsequently, we investigated the association between each of the seven diet-related antioxidants and their respective links to epilepsy overall and its subtypes, including all types, generalized, and focal epilepsy (with documented hippocampal sclerosis), as depicted in Figures 2–4. Furthermore, we examined the relationship between vitamin A (retinol), vitamin C (ascorbic acid), vitamin E (α-tocopherol), vitamin E (γ-tocopherol), carotenoids, and selenium levels in relation to overall epilepsy and various subtypes, as indicated in Supplementary Tables S2–S7. However, we did not observe a significant association between these antioxidants and epilepsy. Little evidence was found to support causal effects of other diet-derived antioxidants on epilepsy.

**Table 2:** Two-sample Mendelian randomization estimations showing the effects of diet-derived antioxidants on the risk of epilepsy and the estimations of heterogeneity and horizontal pleiotropy for results.

Two sample Mendelian randomization					Heterogeneity		Pleiotropy	
Outcome	Exposure	Method	OR (95% CI)	*p*-value	*Q*-statistic	*p*-value	Egger Intercept	*p*-value
All epilepsy	Zinc	Inverse variance weighted	1.06 (1.02–1.11)	0.01^*^	4.27	0.37		
		Weighted median	1.07 (1.01–1.14)	0.02^*^				
		MR Egger	1.06 (0.86–1.32)	0.61	4.27	0.23	0	0.99
Generalized epilepsy	Zinc	Inverse variance weighted	1.12 (1.01–1.25)	0.03^*^	7.77	0.10		
		Weighted median	1.06 (0.96–1.18)	0.27				
		MR Egger	1.03 (0.63–1.68)	0.92	7.44	0.06	0.02	0.74
Focal epilepsy	Zinc	Inverse variance weighted	1.04 (0.98–1.09)	0.17	2.74	0.60		
		Weighted median	1.04 (0.98–1.11)	0.23				
		MR Egger	1.04 (0.84–1.29)	0.75	2.74	0.43	0	0.99
JME	Zinc	Inverse variance weighted	1.00 (0.98–1.01)	0.52	8.11	0.15		
		Weighted median	0.99 (0.98–1.00)	0.13				
		MR Egger	0.97 (0.93–1.00)	0.14	4.7	0.32	0.01	0.16
JAE	Zinc	Inverse variance weighted	1.00(1.00–1.01)	0.10	1.71	0.89		
		Weighted median	1.01 (1.00–1.01)	0.12				
		MR Egger	1.00 (0.98–1.03)	0.69	1.71	0.79	0	0.98
CAE	Zinc	Inverse variance weighted	1.01 (1.00–1.02)	0.12	6.16	0.29		
		Weighted median	1.01 (0.99–1.02)	0.32				
		MR Egger	0.99 (0.96–1.02)	0.61	4.86	0.30	0	0.36
Focal epilepsy (documented hippocampal sclerosis)	Zinc	Inverse variance weighted	1.01 (1.00–1.02)	0.03^*^	6.70	0.24		
		Weighted median	1.01 (1.00–1.02)	0.01^*^				
		MR Egger	1.03 (1.01–1.06)	0.07	3.58	0.47	0	0.15
Focal epilepsy (documented lesion negative)	Zinc	Inverse variance weighted	1.01 (1.00–1.03)	0.06	6.73	0.24				
		Weighted median	1.02 (1.00–1.04)	0.048^*^						
		MR Egger	1.04 (0.98–1.10)	0.26	5.72	0.22	0	0.45
Focal epilepsy (documented lesion other than hippocampal sclerosis)	Zinc	Inverse variance weighted	1.00 (0.99–1.02)	0.60	2.93	0.71		
		Weighted median	1.00 (0.98–1.02)	1.00				
		MR Egger	1.00 (0.96–1.05)	0.84	2.92	0.57	0	0.95
Generalized epilepsy with tonic-clonic seizures	Zinc	Inverse variance weighted	1.00 (1.00–1.00)	0.82	3.38	0.64		
		Weighted median	1.00 (0.99–1.01)	0.92				
		MR Egger	1.00 (0.98–1.01)	0.95	3.37	0.50	0	0.90

OR, odds ratio; CI, confidence interval. *p-value <0.05.

### 3.3 Sensitivity analysis

Cochrane’s *Q* test revealed heterogeneity in vitamin A (retinol) across all types of epilepsy and focal epilepsy (refer to Supplementary Table S5). Significant heterogeneity was observed in vitamin C (ascorbate) for generalized epilepsy and Juvenile Myoclonic Epilepsy (JME) (*p* < 0.05 in IVW and MR-Egger regression) (refer to Supplementary Table S4). Vitamin E (α-tocopherol) exhibited significant heterogeneity across all types of epilepsy, generalized epilepsy, focal epilepsy, and focal epilepsy (documented lesion other than hippocampal sclerosis) (*p* < 0.05 in IVW and MR-Egger regression) (refer to Supplementary Table S2). Vitamin E (γ-tocopherol) showed heterogeneity in generalized epilepsy and Juvenile Absence Epilepsy (JAE) (refer to Supplementary Table S3). Selenium demonstrated heterogeneity in focal epilepsy (documented hippocampal sclerosis) (refer to Supplementary Table S7). Additionally, MR-Egger regression revealed no signs of directional pleiotropy for any outcomes. After excluding outliers, MR-Egger exhibited pleiotropic *p*-values >0.05 (refer to Supplementary Tables S2–S7).

## 4 Discussion

In our study, we conducted a two-sample MR analysis to investigate the relationship between diet-derived antioxidants and epilepsy occurrence. Our findings revealed a clear association between genetically increased levels of circulating zinc and epilepsy. However, we did not find significant correlations between epilepsy and genetically elevated levels of other common antioxidants, including circulating ascorbate, α-tocopherol, γ-tocopherol, carotene, retinol, and selenium.

Epilepsy, a neurological disorder characterized by a persistent tendency to experience seizures (33), can lead to neuronal death and promote further seizures (34–36). Processes involved in epileptogenesis include changes in neuroinflammation, synapses, neurotransmitters, receptors, oxidative stress, mitochondrial dysfunction, cytokine signaling, and apoptosis (37–39). Oxidative stress plays a crucial role in epilepsy development, with emerging research increasingly supporting a connection between epilepsy and heightened production of reactive oxygen species (ROS) production. Seizures can trigger the production of reactive oxygen/nitrogen species (ROS/RNS), leading to oxidative stress and cellular damage (40, 41). At the same time, the production of reactive substances or impaired activity of the antioxidant system underlies various forms of epilepsy, thus increasing the risk of recurrent seizures (42, 43). Both clinical and experimental studies suggest that oxidative stress plays a dual role as both a cause and consequence in the progression of epilepsy (5, 6). Elevated levels of oxidative stress biomarkers are associated with various models of epilepsy (44). Therefore, inhibiting the production of oxidative stress is expected to be a therapeutic window of opportunity for seizure prevention.

Numerous studies have explored the therapeutic potential of antioxidant compounds in managing epilepsy. Dietary interventions can modify underlying physiological processes and positively impact clinical outcomes. Historically, dietary modifications have been investigated as potential treatments for epilepsy. Research has identified several nutrients with anti-inflammatory or antioxidant properties, such as vitamin A, vitamin C, omega-3 fatty acids, polyphenols, and carotenoids (45–50). Vitamin E helps to remove ROS from the body and protects lipids and proteins from oxidative damage (51, 52). It has potential as a continuous adjuvant treatment for refractory epilepsy (53, 54). Antioxidant defense mechanisms also involve Zinc (Zn^2+^) (55) and Selenium (Se^2+^) (56). Unfortunately, only a few antioxidant-containing therapeutic interventions have been thoroughly studied as supplementary therapy for epilepsy patients, and these studies only partially succeeded in evaluating their prospective benefits. High-quality data from direct clinical research on the application of antioxidants in epileptic patients are scarce.

Our study found that higher blood zinc levels increased the risk of seizures in specific epilepsy subtypes, including all types of epilepsy, generalized epilepsy, and focal epilepsy (documented hippocampal sclerosis). Previous studies suggest that zinc exhibits a biphasic response in the central nervous system, with both neurotoxic and neuroprotective effects depending on its concentration (57, 58). In epilepsy, zinc shows both pro and anti-convulsant effects (58–60). For patients with these three seizure types, it is crucial to monitor and manage their blood zinc levels to reduce the risk of zinc-induced seizures. Maintaining zinc homeostasis appears to contribute to its anti-epileptic effect (61). Moderate zinc levels, when combined with traditional anti-epileptic drugs, acts synergistically (62). Thus, supplementing with moderate amounts of zinc to maintain balanced levels may be effective (61). However, standardized zinc supplement intake and optimal blood zinc concentrations have yet to be established. This will be a focus of our future research. Meanwhile, our findings suggest that most other diet-derived circulating antioxidants, including ascorbate, α-tocopherol, γ-tocopherol, carotene, retinol, and selenium, are unlikely to have a causal association with the risk of epilepsy.

### 4.1 Study strengths

Our study design effectively mitigated residual confounding, addressed issues of reverse causality, and strengthened causal inferences regarding the associations between exposure and epilepsy. Despite a limited number of robust genetic instruments, the study demonstrated a strong capacity for investigating causality due to the minimal overlap between exposure and outcome data. The current literature on the efficacy of diet-derived antioxidants in epilepsy treatment is notably limited. Our MR analysis elucidated the enduring impact of elevated levels of diet-derived antioxidants, accounting for long-term risks unaffected by dietary supplementation. Notably, MR, unlike randomized controlled trials (RCTs), does not require direct subject exposure to antioxidants, allowing it to be implemented at any time without extensive time and resource demands. This approach also mitigates the potential for subjecting individuals to unnecessary risks and harms (63).

### 4.2 Study limitations

First, Cochran’s *Q* values in the MR analysis indicated heterogeneity for some exposures. Second, due to the statistical limitations of the published data we used, we were unable to test for a nonlinear causal association between antioxidant levels and epilepsy. Third, although some findings did not show a causal association with epilepsy, we cannot entirely exclude the possibility that the effect size was too small for detection. Finally, we restricted our analysis to individuals of European ancestry only.

## 5 Conclusion

This study establishes a significant foundation for evaluating the correlation between dietary-derived circulating antioxidants and epilepsy. Our study revealed that higher blood zinc levels were associated with an increased risk of seizures in specific epilepsy subtypes. However, we found no evidence supporting the effects of dietary-derived vitamin A, vitamin C, vitamin E and selenium on epilepsy risk in the general population. Our future research will focus on further exploring the relationship between zinc levels and seizure risk in epilepsy.

## Data availability statement

The datasets presented in this study can be found in online repositories. The names of the repository/repositories and accession number(s) can be found in the article/Supplementary material.

## Ethics statement

Ethical review and approval was not required for the study on human participants in accordance with the local legislation and institutional requirements. Written informed consent from the patients/participants or patients/participants’ legal guardian/next of kin was not required to participate in this study in accordance with the national legislation and the institutional requirements.

## Author contributions

SH: Conceptualization, Data curation, Formal analysis, Methodology, Resources, Software, Writing – original draft. YC: Data curation, Formal analysis, Resources, Software, Writing – original draft. YW: Data curation, Formal analysis, Project administration, Resources, Writing – original draft. SP: Data curation, Methodology, Resources, Software, Writing – original draft. YL: Data curation, Investigation, Methodology, Resources, Writing – original draft. WG: Project administration, Software, Supervision, Validation, Writing – original draft. XH: Funding acquisition, Project administration, Supervision, Validation, Writing – review & editing. QF: Funding acquisition, Methodology, Project administration, Validation, Visualization, Writing – review & editing.
